# The Statistical Fragility of Operative vs Nonoperative Management for Achilles Tendon Rupture: A Systematic Review of Comparative Studies

**DOI:** 10.1177/10711007221108078

**Published:** 2022-08-24

**Authors:** Nathan P. Fackler, Theofilos Karasavvidis, Cooper B. Ehlers, Kylie T. Callan, Wilson C. Lai, Robert L. Parisien, Dean Wang

**Affiliations:** 1 University of California, Irvine, CA, USA; 2 Georgetown University School of Medicine, Washington, DC, USA; 3 University of California, San Diego, CA, USA; 4 Icahn School of Medicine at Mount Sinai, New York, NY, USA

**Keywords:** fragility index, fragility quotient, statistical significance, *P* value, Achilles tendon, operative, nonoperative

## Abstract

**Background:**

The statistical significance of randomized controlled trials (RCTs) and comparative studies is often conveyed utilizing the *P* value. However, *P* values are an imperfect measure and may be vulnerable to a small number of outcome reversals to alter statistical significance. The interpretation of the statistical strength of these studies may be aided by the inclusion of a Fragility Index (FI) and Fragility Quotient (FQ). This study examines the statistical stability of studies comparing operative vs nonoperative management for Achilles tendon rupture.

**Methods:**

A systematic search was performed of 10 orthopaedic journals between 2000 and 2021 for comparative studies focusing on management of Achilles tendon rupture reporting dichotomous outcome measures. FI for each outcome was determined by the number of event reversals necessary to alter significance (*P* < .05). FQ was calculated by dividing the FI by the respective sample size. Additional subgroup analyses were performed.

**Results:**

Of 8020 studies screened, 1062 met initial search criteria with 17 comparative studies ultimately included for analysis, 10 of which were RCTs. A total of 40 outcomes were examined. Overall, the median FI was 2.5 (interquartile range [IQR] 2-4), the mean FI was 2.90 (±1.58), the median FQ was 0.032 (IQR 0.012-0.069), and the mean FQ was 0.049 (±0.062). The FI was less than the number of patients lost to follow-up for 78% of outcomes.

**Conclusion:**

Studies examining the efficacy of operative vs nonoperative management of Achilles tendon rupture may not be as statistically stable as previously thought. The average number of outcome reversals needed to alter the significance of a given study was 2.90. Future analyses may benefit from the inclusion of a fragility index and a fragility quotient in their statistical analyses.

**Level of Evidence:** Level II, systematic review of Level I and Level II studies.

## Introduction

The Achilles tendon is the most commonly ruptured tendon in the lower extremity, with an increasing annual reported incidence for acute Achilles tendon ruptures of up to 40 per 100 000/year.^19,24,37^ Treatment options include nonsurgical management with the use of a cast-boot or functional brace and surgical repair of the tendon.^59^ Several randomized controlled trials (RCTs) have sought to investigate the differences between operative and nonoperative options, with many trials showing no differences in patient-reported outcomes and rerupture rates.^43,59,65^ The American Academy of Orthopaedic Surgeons have yet to make a strong recommendation in favor of either operative or nonoperative management, and as such there remains a substantial practice variation among surgeons for this injury.^15,59^

The *P* value is a commonly used statistical tool to evaluate outcomes in research. When the *P* value is less than the threshold value, typically .05, the null hypothesis is rejected, indicating that there is a less than 5% chance that the difference measured occurred because of random chance.^4,16,63^ This scenario is further interpreted as representing a “statistically significant” event. However, the *P* value is vulnerable to pitfalls in study design and study power as it does not account for effect size, strength of association, or applicability of an outcome to a specific population.^25,63^ Furthermore, 96% of MEDLINE articles containing *P* values report at least 1 with a value of .05 or less. This is likely due to a variety of factors including, but not limited to, multiple testing, *P*-hacking, publication bias, and underpowered studies.^2,7,46^ To this end, there is concern among medical professionals that the .05 threshold may be arbitrary or inappropriate and that its sole use for the statistical interpretation of a study may not be adequate.

Therefore, the Fragility Index (FI) has recently been introduced as a complement to traditional statistical analyses as represented by *P* values. FI is calculated from dichotomous outcomes by reversing the outcome status of patients included in one study arm, with the goal of determining the minimum number of outcome event reversals necessary to switch a finding from statistically significant to not statistically significant, or vice versa.^15,63^ A large FI conveys to the reader more confidence in the statistical strength of a study outcome, suggesting that the reversal of a relatively large number of events is required to alter the observed result. The relevance of the FI is based on sample size and can therefore vary in strength depending on the power of the study. For example, an FI of 10 carries more weight in a smaller cohort study with a total of 50 patients as opposed to a larger population database study with 50 000 patients. Consequently, there is no specific threshold for FI to indicate the robustness of a study.^29^ To address this issue, the Fragility Quotient (FQ) was introduced, dividing the FI by the sample size to achieve a value of relative stability. As such, the FQ demonstrates the percentage of reversals required to alter statistical significance, and therefore, statistical stability is most effectively communicated through the inclusion of both FI and FQ values.^1,15^

The published literature investigating the statistical robustness of comparative studies via the utilization of fragility analysis has demonstrated relatively low FI and FQ values, with multiple studies reporting FIs ranging from 2 to 5, a number that is usually less than the number of patients lost to follow-up.^3,20,26,28,32,35,39–41,43,45,54,61,62,64,65^ Thus, the significance of a result could be altered by simply maintaining patient follow-up.^63^ To date, no studies have used FI and FQ to evaluate the literature relevant to operative vs nonoperative management of Achilles tendon ruptures. The purpose of the present study is to determine the statistical stability of studies comparing operative to nonoperative management for Achilles tendon rupture. The primary objective was to calculate the FI and FQ for dichotomous outcome measures, including tendon rerupture, of the included studies. The secondary aim was to conduct subgroup analysis to determine the proportion of outcome events for which FI was fewer than the number of patients lost to follow-up (LTF). The authors hypothesize that more than half of outcomes analyzed will have a loss to follow-up greater than the fragility index for that outcome.

## Methods

Comparative studies and RCTs comparing outcomes of operative vs nonoperative management of Achilles tendon ruptures published in select journals from 2000 to 2021 were identified and collected. The journals were selected for their prominence within the field of orthopaedic surgery and foot and ankle surgery. The 10 orthopaedic journals included were *British Journal of Sports Medicine* (*BJSM*), *American Journal of Sports Medicine* (*AJSM*), *Journal of Bone & Joint Surgery* (*JBJS*), *Clinical Orthopaedics and Related Research* (*CORR*), *Bone & Joint Journal, Sports Health Journal, International Orthopaedics, Knee Surgery, Sports Traumatology, Arthroscopy* (*KSSTA*), *Foot & Ankle International* (*FAI*), and *Foot and Ankle Surgery*. According to the 2020 InCites Journal Citation Reports index, these journals are recognized as the most impactful in the field of orthopaedic and foot and ankle surgery with impact factors of 12.022, 5.810, 4.578, 4.329, 4.306, 3.843, 2.854, 2.728, 2.292, and 1.776, respectively.^8^

**Figure 1.**
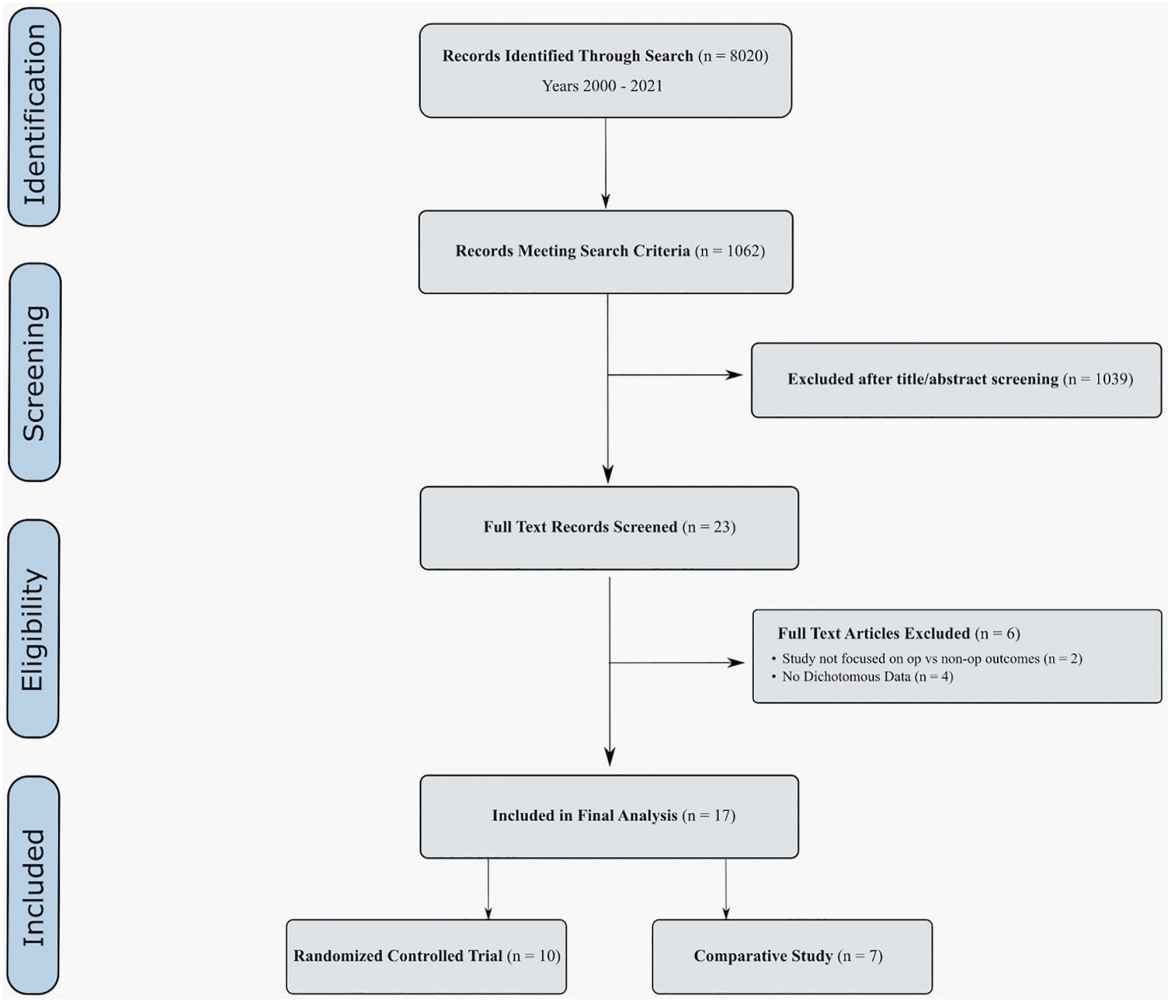
Study identification flowchart.

Studies from these journals were reviewed in adherence to the Preferred Reporting Items for Systematic Reviews and Meta-Analyses (PRISMA) guidelines.^33^ Initial PubMed search was conducted by searching by “Journal” and then utilizing the “AND” tool to search for all articles containing the words *Achilles, gastrocnemius*, or *soleus*. For example, the search in *Foot & Ankle International* was as follows: ((("Foot ankle international"[Journal]) AND (achilles)) OR (gastrocnemius)) OR (soleus). The titles and abstracts of these studies were then screened independently by 2 authors (NF, CE). Any disagreements in article selection that arose were settled by the senior author (DW). Included studies compared operative vs nonoperative management of Achilles tendon ruptures. The studies were excluded if (1) the surgical technique was not explicitly described or referenced; (2) patients with an incomplete Achilles tendon tear were included; (3) the patients underwent revision Achilles tendon repair; (4) the studies were cadaveric, in vitro, or animal studies; (5) the study used population databases, national registries, or cross-sectional data; (6) no dichotomous outcomes were reported anywhere in the study; and (7) the study was not related to operative vs nonoperative outcomes (blood loss, anesthesia time, etc). From the studies meeting these criteria, all categorical outcomes were included. Nondichotomous data points were not included as these are unable to be analyzed with current fragility methodology (Figure 1).

The quality of included studies was assessed independently by 2 authors (NF, WL) using the Cochrane Risk of Bias for Randomized Trials (ROB-2) tool and Methodological Index for Non-Randomized Studies (MINORS) criteria for randomized and nonrandomized studies, respectively. The ROB-2 tool examines risk of bias under 5 domains: (1) randomization process, (2) deviations from intended intervention, (3) missing data, (4) measurement of the outcome, (5) selection of the reported result. Each article is assessed and assigned a score of low risk, some concerns, or high risk of bias for each domain.^30^ MINORS is a validated scoring system for nonrandomized studies that gives a score of 0, 1, or 2 to 12 criteria assessing bias for a maximum score of 24 for comparative studies.^58^

Data involving dichotomous outcomes were extracted from each study including the number of patients in each outcome group, the outcome being measured, total population size, and the number lost to follow-up. The reported *P* value associated with each dichotomous outcome measure was recorded and verified for accuracy using a Fisher exact test. Statistical significance was set as a *P* value <.05. Using a contingency table, the results of the outcomes were manipulated until the significance was reversed. For example, if the *P* value of a certain outcome was reported as less than .05, the number of outcome reversals needed to increase the *P* value above .05 was determined, and vice versa. FI was recorded as the number of outcome reversals needed to change the significance of the study. FQ was determined by dividing the FI by the respective sample size. Studies whose FI was less than their number lost to follow-up were identified. Six subgroups were analyzed for significant differences via independent *t* tests at 95% confidence: (1) significant (*P* < .05) vs insignificant (*P* > .05) outcomes, (2) outcomes for which the FI was fewer than the number of patients lost to follow-up vs outcomes for which the FI was greater than the number of patients lost to follow-up, (3) outcomes between rates of rerupture and all other outcomes, (4) outcomes from RCTs vs those from nonrandomized comparative studies (5) Primary outcomes vs secondary outcomes, and (6) outcomes from studies determined to be low risk of bias by the ROB-2 tool (ie, high-quality studies) vs outcomes from all other studies. Data analysis was performed in Microsoft Excel (version 16.37).

## Results

Of the 8020 studies identified, 1062 comparative studies were screened. Ultimately, 17 studies were included for the analysis, including 10 RCTs. Details of the included studies can be found in Appendix 1.

**Figure 2.**
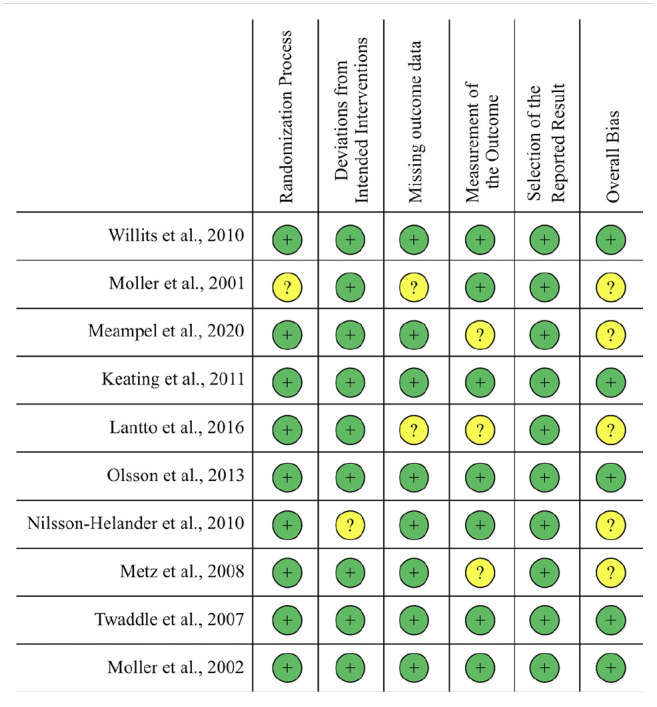
Outcomes of the Cochrane Risk Of Bias 2.0 Tool.^60^ The plus sign indicates a low risk of bias, and the question mark indicates that there is some concern for bias.

A summary of risk of bias for randomized studies utilizing the ROB-2 tool is shown in Figure 2, and MINORS criteria scoring for nonrandomized studies is demonstrated in Table 1. Five of the 10 RCTs had some concern for risk of bias found in their study. The average MINORS score for comparative studies was 14 (range 13-16).

**Table 1.** MINORS Scores for Nonrandomized Comparative Studies.^a^

Primary Author	Bergkvist^3^	Lim^34^	Renninger^54^	Gwynne-Jones^20^	Jaakkola^26^	Westin^64^	van der Linden-van der Zwaag^62^
A clearly stated aim	2	2	2	2	2	2	2
Inclusion of consecutive patients	2	1	2	2	2	2	2
Prospective collection of data	0	0	0	0	0	0	0
Endpoints appropriate to the aim of the study	2	2	2	2	1	2	2
Unbiased assessment of the study endpoint	0	0	0	0	0	0	1
Follow-up period appropriate to the aim of the study	2	2	0	1	2	2	1
Loss to follow-up less than 5%	0	0	0	0	0	0	0
Prospective calculation of the study size	0	0	0	0	0	1	0
An adequate control group	2	2	2	2	2	2	2
Contemporary groups	2	0	1	2	2	2	1
Baseline equivalence of groups	1	2	2	0	1	1	1
Adequate statistical analyses	2	2	2	2	2	2	2
Total MINORS score	15	13	13	13	14	16	14

a Values reported as a number out of a possible 24 points.

A total of 40 dichotomous outcomes from the 17 studies examined were analyzed. Across all outcomes, the median FI was 2.5 (interquartile range [IQR] 2-4), the median FQ was 0.032 (IQR 0.012-0.069), the mean FI was 2.9 (±1.58), and the mean FQ was 0.049 (±0.061). Across all studies, with the mean FI and FQ of each study weighted evenly, mean FI was 2.81 (±1.31) and mean FQ was 0.040 (±0.028). The FI was greater than the number lost to follow-up (LTF) for 78% of outcomes. The results of the subgroup analysis can be found in Table 2.

**Table 2.** Fragility for Analyzed Subgroups.

Characteristic	Outcomes	Mean FI (SD)	Median FI (IQR)	Mean FQ (SD)	Median FQ (IQR)
All trials	40	2.90 (1.58)	2.5 (2-4)	0.049 (0.061)	0.032 (0.012-0.069)
Reported *P* value					
*P* < .05	6	2.33 (1.51)	2.0 (1-4)	0.022 (0.030)	0.006 (0.003-0.057)
*P* > .05	34	3.00 (1.60)	2.5 (2-4)	0.054 (0.065)	0.033 (0.018-0.070)
*P* value		.17		.12	
Lost to follow-up					
FI < LTF	31	2.77 (1.54)	2.0 (2-4)	0.052 (0.068)	0.032 (0.012-0.070)
FI > LTF	9	3.30 (1.73)	4.0 (1.5-4.5)	0.042 (0.033)	0.037 (0.011-0.061)
*P* value–		.18		.34	
Randomization					
RCT	22	2.91 (1.27)	3.0 (2-4)	0.043 (0.025)	0.032 (0.027-0.058)
Non-RCT	18	2.89 (1.94)	2.0 (1-5)	0.057 (0.088)	0.018 (0.004-0.088)
*P* value		.48		.24	
Outcome reported as rerupture					
Rerupture	15	3.07 (1.75)	3.0 (1-4)	0.035 (0.029)	0.036 (0.006-0.080)
All other outcomes	25	2.80 (1.50)	2.0 (2-4)	0.058 (0.074)	0.032 (0.014-0.059)
*P* value–		.31		.13	
Primary vs secondary outcome					
Primary	14	3.29 (1.82)	3.5 (1.5-5)	0.043 (0.039)	0.039 (0.009-0.093)
Secondary	26	2.69 (1.44)	2.0 (1.5-4)	0.053 (0.071)	0.048 (0.024-0.105)
*P* value		.13		.33	
Studies at low risk of bias vs all others					
Low risk	7	3.71 (1.25)	4.0 (4-4)	0.061 (0.125)	0.053 (0.039-0.089)
All others	33	2.73 (1.61)	2.0 (1.5-4)	0.047 (0.066)	0.031 (0.011-0.059)
*P* value		.07		.29	

Abbreviations: FI, Fragility Index; FQ, Fragility Quotient; IQR, interquartile range; LTF, lost to follow-up; RCT, randomized controlled trial.

No significant differences were found across any of the subgroups analyzed. The largest difference found in the subgroup analysis was the FI of outcomes in studies with no concern for risk of bias (3.71 ± 1.25) compared to outcomes in all other studies (2.73 ± 1.61) (*P* = .07). The next largest differences were found in the FQ of significant (*P* < .05) outcomes (0.022 ± 0.030) compared to insignificant (*P* > .05) outcomes (0.054 ± 0.065, *P* = .113), and the FQ of rerupture (0.035 ± 0.029) compared to all other outcomes (0.058 ± 0.074, *P* = .133).

## Discussion

For the outcomes included in this analysis, the median FI was 2.5 (IQR 2-4), the mean FI was 2.9 (±1.58), the median FQ was 0.032 (IQR 0.012-0.069), and the mean FQ was 0.049 (±0.062). These values are consistent with the current orthopaedic literature reporting an average median FI of 3.81^10–14,17,18,21,23,29–31,36,38,42,47–53,55–57^ and FQ of 0.048.^10–12,18,21,23,31,47–51,53^ The hypothesis of this study was confirmed, with more than half (78%) of outcomes analyzed having a loss to follow-up greater than the fragility index for that outcome. Furthermore, outcomes from studies with a greater risk of bias and rerupture as an individual outcome were more fragile than the other outcomes observed in this analysis.

This study expands on a discussion started by a recent fragility analysis examining Achilles tendon injury in top orthopaedic journals.^48^ In their review, Parisien et al analyzed outcomes across studies focusing on Achilles tendon injury and found that these data lacked statistical stability. The current study narrowed its focus on a specific clinical question: operative vs nonoperative management of Achilles tendon rupture. This analysis revealed that outcomes in operative vs nonoperative studies were more fragile (median FI = 2.9) than the overall literature on Achilles tendon injury (median FI = 4). Furthermore, LTF >FI was found to be higher in the studies included in this analysis (78%) compared with Achilles tendon injury literature (70.5%).^48^ The findings from this study add to the growing body of evidence supporting the inclusion of fragility indices and quotients in studies focused on Achilles tendon rupture management and the orthopaedic literature as a whole.

A recent systematic review and meta-analysis examined many of the trials included in this study and concluded that surgery decreases risk of rerupture but increases overall risk of complications related to surgery, and that the choice of operative vs nonoperative management should be patient specific.^44^ Multiple reviews have noted that heterogeneity among rehabilitation protocols, timing of weightbearing status, and duration of follow-up can all contribute to the lack of consensus regarding which treatment modality is superior.^27,44,59^ There is also significant heterogeneity among surgical repair strategies, including traditional open vs minimally invasive techniques and use of suture anchors and biologics. Ultimately, future high-quality research examining each of these factors in both active and sedentary populations will be necessary to further delineate any differences in outcomes between operative and nonoperative treatment of Achilles tendon ruptures. The results of this study place an increased emphasis on the need for high-quality research on the topic, as it has been demonstrated that high-quality studies are less fragile than studies with a greater risk of bias.

The fragility index has received some criticism recently, with some calling it a *P* value in disguise^6^ and an oversimplification of the complex, nonlinear relationships between various factors in a given study.^9^ Indeed, the fragility index is an offshoot of the *P* value and therefore should be taken as a metric to aide in the interpretation of the *P* value.^22^ Other important metrics of a study’s robustness such as study design, prospective sample size calculations, preregistration of planned analyses, and transparent reporting of procedures and statistical analyses should all be taken into consideration when interpreting the results of a study. The inclusion of FI and FQ in a given analysis should be viewed as an additional tool in the clinician’s arsenal for the interpretation of the statistical conclusions of a study.

This study should be interpreted within the context of its limitations. First, FI and FQ can only be calculated from outcomes using dichotomous data, and therefore, the fragility of important continuous variables such as muscle dynamometry and Short Musculoskeletal Function Assessment scores cannot be determined with this mode of analysis. Future analyses examining continuous outcomes using the method developed recently by Caldwell et al^5^ would be beneficial for the literature. Because only dichotomous outcomes could be analyzed, 4 studies were excluded. This study examined outcomes from articles published in the top 10 highest-impact journals in sports and foot and ankle surgery. This may be considered both a strength and a weakness as the data from these high-impact journals represent some of the best evidence available on the topic; however, there is potential for other studies to be published outside of these selected journals that were not included in this analysis. Finally, although having a majority high-quality RCTs in this analysis may be considered a strength, the heterogeneity of included studies, both in surgical technique and in patient population studied may be considered a weakness of this analysis.

## Conclusion

The statistical significance of studies examining the operative vs nonoperative management of Achilles tendon ruptures is fragile. In particular, outcomes from studies with greater risk of bias proved to be more fragile than the rest of the literature. A focus on high-quality, statistically robust analyses of operative vs nonoperative management of Achilles tendon rupture will minimize this risk of fragility in the future. These future studies may benefit from the inclusion of an FI and FQ in their statistical analyses.

## Supplemental Material

sj-docx-1-fai-10.1177_10711007221108078 – Supplemental material for The Statistical Fragility of Operative vs Nonoperative Management for Achilles Tendon Rupture: A Systematic Review of Comparative Studies Click here for additional data file. Supplemental material, sj-docx-1-fai-10.1177_10711007221108078 for The Statistical Fragility of Operative vs Nonoperative Management for Achilles Tendon Rupture: A Systematic Review of Comparative Studies by Nathan P. Fackler, Theofilos Karasavvidis, Cooper B. Ehlers, Kylie T. Callan, Wilson C. Lai, Robert L. Parisien and Dean Wang in Foot & Ankle International

## References

[1] Ahmed W Fowler RA McCredie VA. Does sample size matter when interpreting the fragility index? Crit Care Med. 2016;44(11):e1142-e1143. doi:10.1097/CCM.0000000000001976. 27755081

[2] Benjamin DJ Berger JO Johannesson M , et al. Redefine statistical significance. Nat Hum Behav. 2017;2(1):6-10. doi:10.1038/s41562-017-0189-z. 30980045

[3] Bergkvist D Åström I Josefsson P Dahlberg L. Acute Achilles tendon rupture: a questionnaire follow-up of 487 patients. J Bone Joint Surg Am. 2012;94(13):1229-1233. doi:10.2106/JBJS.J.01601. 22760392

[4] Biau DJ Jolles BM Porcher R. P value and the theory of hypothesis testing: An explanation for new researchers. Clin Orthop Relat Res. 2010;468(3):885-892. doi:10.1007/s11999-009-1164-4. 19921345 PMC2816758

[5] Caldwell JME Youssefzadeh K Limpisvasti O . A method for calculating the fragility index of continuous outcomes. J Clin Epidemiol. 2021;136:20-25. doi:10.1016/J.JCLINEPI.2021.02.023. 33684509

[6] Carter RE McKie PM Storlie CB. The Fragility Index: a P-value in sheep’s clothing? Eur Heart J. 2017;38(5):346-348. doi:10.1093/EURHEARTJ/EHW495. 28417139

[7] Chavalarias D Wallach JD Li AHT Ioannidis JPA . Evolution of reporting P values in the biomedical literature, 1990-2015. JAMA. 2016;315(11):1141-1148. doi:10.1001/jama.2016.1952. 26978209

[8] Clarivate Analytics. Journal Citation Reports. Clarivate Analytics; 2020.

[9] Condon TM Sexton RW Wells AJ To MS. The weakness of fragility index exposed in an analysis of the traumatic brain injury management guidelines: a meta-epidemiological and simulation study. PLoS One. 2020;15(8):e0237879. doi:10.1371/JOURNAL.PONE.0237879. PMC7433866 32810192

[10] Constant M Trofa DP Saltzman BM Ahmad CS Li X Parisien RL. The fragility of statistical significance in patellofemoral instability research: a systematic review. Am J Sports Med. Published online October 11, 2021. doi:10.1177/03635465211039202. 34633219

[bibr11-10711007221108078] Ehlers CB Curley AJ Fackler NP , et al. The statistical fragility of single-bundle vs double-bundle autografts for ACL reconstruction: a systematic review of comparative studies. Orthop J Sport Med. 2021;9(12):232596712110646. doi:10.1177/23259671211064626. PMC8721389 34988239

[bibr12-10711007221108078] Ehlers CB Curley AJ Fackler NP Minhas A Chang ES. The statistical fragility of hamstring versus patellar tendon autografts for anterior cruciate ligament reconstruction: a systematic review of comparative studies. Am J Sports Med. 2021;49(10):2827-2833. doi:10.1177/0363546520969973. 33211555

[bibr13-10711007221108078] Ekhtiari S Gazendam A Nucci N Kruse C Bhandari M. The fragility of statistically significant findings from randomized controlled trials in hip and knee arthroplasty. J Arthroplasty. 2021;36(6):2211-2218.e1. doi:10.1016/J.ARTH.2020.12.015. 33390336

[bibr14-10711007221108078] Evaniew N Files C Smith C , et al. The fragility of statistically significant findings from randomized trials in spine surgery: a systematic survey. Spine J. 2015;15(10):2188-2197. doi:10.1016/j.spinee.2015.06.004. 26072464

[bibr15-10711007221108078] Feinstein AR. The unit fragility index: an additional appraisal of “statistical significance” for a contrast of two proportions. J Clin Epidemiol. 1990;43(2):201-209. doi:10.1016/0895-4356(90)90186-S. 2303850

[bibr16-10711007221108078] Fisher RA . Statistical Methods for Research Workers. In: Kotz S Johnson NL , eds. Breakthroughs in Statistics. Springer Series in Statistics. Springer; 1992:66-70. doi:10.1007/978-1-4612-4380-9_6.

[bibr17-10711007221108078] Forrester L Jang E Lawson M Capi A Tyler W. Statistical fragility of surgical and procedural clinical trials in orthopaedic oncology. J Am Acad Orthop Surg Glob Res Rev. 2020;4(6):e19.00152. doi:10.5435/JAAOSGLOBAL-D-19-00152. PMC7322779 32656478

[bibr18-10711007221108078] Forrester LA McCormick KL Bonsignore-Opp L , et al. Statistical fragility of surgical clinical trials in orthopaedic trauma. J Am Acad Orthop Surg Glob Res Rev. 2021;5(11): e20.00197. doi:10.5435/JAAOSGLOBAL-D-20-00197. PMC8608260 34807889

[bibr19-10711007221108078] Ganestam A Kallemose T Troelsen A Barfod K. Increasing incidence of acute Achilles tendon rupture and a noticeable decline in surgical treatment from 1994 to 2013. A nationwide registry study of 33,160 patients. Knee Surg Sports Traumatol Arthrosc. 2016;24(12):3730-3737. doi:10.1007/S00167-015-3544-5. 25697284

[bibr20-10711007221108078] Gwynne-Jones D Sims M Handcock D. Epidemiology and outcomes of acute Achilles tendon rupture with operative or nonoperative treatment using an identical functional bracing protocol. Foot Ankle Int. 2011;32(4):337-343. doi:10.3113/FAI.2011.0337. 21733434

[bibr21-10711007221108078] Herndon CL McCormick KL Gazgalis A Bixby EC Levitsky MM Neuwirth AL. Fragility index as a measure of randomized clinical trial quality in adult reconstruction: a systematic review. Arthroplast Today. 2021;11:239. doi:10.1016/J.ARTD.2021.08.018. 34692962 PMC8517286

[bibr22-10711007221108078] Ho AK. The Fragility Index for assessing the robustness of the statistically significant results of experimental clinical studies. J Gen Intern Med. 2022;37(1):206-211. doi:10.1007/S11606-021-06999-9/FIGURES/2. 34357573 PMC8739402

[bibr23-10711007221108078] Huang X Chen B Thabane L Adachi JD Li G. Fragility of results from randomized controlled trials supporting the guidelines for the treatment of osteoporosis: a retrospective analysis. Osteoporos Int. 2021;32(9):1713-1723. doi:10.1007/s00198-021-05865-y. 33595680

[bibr24-10711007221108078] Huttunen T Kannus P Rolf C Felländer-Tsai L Mattila V. Acute achilles tendon ruptures: incidence of injury and surgery in Sweden between 2001 and 2012. Am J Sports Med. 2014;42(10):2419-2423. doi:10.1177/0363546514540599. 25056989

[bibr25-10711007221108078] Ioannidis JPA . Contradicted and initially stronger effects in highly cited clinical research. J Am Med Assoc. 2005; 294(2):218-228. doi:10.1001/jama.294.2.218. 16014596

[bibr26-10711007221108078] Jaakkola J Beskin J Griffith L Cernansky G. Early ankle motion after triple bundle technique repair vs. casting for acute Achilles tendon rupture. Foot Ankle Int. 2001;22(12):979-984. doi:10.1177/107110070102201210. 11783925

[bibr27-10711007221108078] Jiang N Wang B Chen A Dong F Yu B. Operative versus nonoperative treatment for acute Achilles tendon rupture: a meta-analysis based on current evidence. Int Orthop. 2012; 36(4):765-773. doi:10.1007/S00264-011-1431-3. 22159659 PMC3311794

[bibr28-10711007221108078] Keating J Will E. Operative versus non-operative treatment of acute rupture of tendo Achillis: a prospective randomised evaluation of functional outcome. J Bone Joint Surg Br. 2011;93(8):1071-1078. doi:10.1302/0301-620X.93B8.25998. 21768631

[bibr29-10711007221108078] Khan M Evaniew N Gichuru M , et al. The fragility of statistically significant findings from randomized trials in sports surgery: a systematic survey. Am J Sports Med. 2017;45(9): 2164-2170. doi:10.1177/0363546516674469. 27895038

[bibr30-10711007221108078] Khormaee S Choe J Ruzbarsky JJ , et al. The fragility of statistically significant results in pediatric orthopaedic randomized controlled trials as quantified by the Fragility Index: a systematic review. J Pediatr Orthop. 2018;38(8):e418-e423. doi:10.1097/BPO.0000000000001201. 29979332

[bibr31-10711007221108078] Kyriakides PW Schultz BJ Egol K Leucht P. The fragility and reverse fragility indices of proximal humerus fracture randomized controlled trials: a systematic review. Eur J Trauma Emerg Surg. Published online May 31, 2021. doi:10.1007/s00068-021-01684-2. 34056677

[bibr32-10711007221108078] Lantto I Heikkinen J Flinkkila T , et al. A prospective randomized trial comparing surgical and nonsurgical treatments of acute Achilles tendon ruptures. Am J Sports Med. 2016; 44(9):2406-2414. doi:10.1177/0363546516651060. 27307495

[bibr33-10711007221108078] Liberati A Altman DG Tetzlaff J , et al. The PRISMA statement for reporting systematic reviews and meta-analyses of studies that evaluate healthcare interventions: explanation and elaboration. BMJ. 2009;339:b2700. doi:10.1136/bmj.b2700. PMC2714672 19622552

[bibr34-10711007221108078] Lim C Lees D Gwynne-Jones D. Functional outcome of acute Achilles tendon rupture with and without operative treatment using identical functional bracing protocol. Foot Ankle Int. 2017;38(12):1331-1336. doi:10.1177/1071100717728687. 28891323

[bibr35-10711007221108078] Maempel J Clement N Wickramasinghe N Duckworth A Keating J. Operative repair of acute Achilles tendon rupture does not give superior patient-reported outcomes to nonoperative management. Bone Joint J. 2020;102-B(7):933-940. doi:10.1302/0301-620X.102B7.BJJ-2019-0783.R3. 32600149

[bibr36-10711007221108078] Maldonado DR Go CC Huang BH Domb BG. The fragility index of hip arthroscopy randomized controlled trials: a systematic survey. Arthroscopy. 2021;37(6):1983-1989. doi:10.1016/j.arthro.2021.01.049. 33539980

[bibr37-10711007221108078] Mattila V Huttunen T Haapasalo H Sillanpää P Malmivaara A Pihlajamäki H. Declining incidence of surgery for Achilles tendon rupture follows publication of major RCTs: evidence-influenced change evident using the Finnish registry study. Br J Sports Med. 2015;49(16):1084-1086. doi:10.1136/BJSPORTS-2013-092756. 24128757

[bibr38-10711007221108078] McCormick K Tedesco L Swindell H Forrester L Jobin C Levine W. Statistical fragility of randomized clinical trials in shoulder arthroplasty. J Shoulder Elbow Surg. 2021;30(8): 1787-1793. doi:10.1016/J.JSE.2020.10.028. 33271323

[bibr39-10711007221108078] Metz R Erleisdonk E van der Heijden G , et al. Acute Achilles tendon rupture: minimally invasive surgery versus nonoperative treatment with immediate full weightbearing–a randomized controlled trial. Am J Sports Med. 2008;36(9):1688-1694. doi:10.1177/0363546508319312. 18645042

[bibr40-10711007221108078] Möller M Kälebo P Tidebrant G TMovin T Karlsson J. The ultrasonographic appearance of the ruptured Achilles tendon during healing: a longitudinal evaluation of surgical and nonsurgical treatment, with comparisons to MRI appearance. Knee Surg Sports Traumatol Arthrosc. 2002;10(1):49-56. doi:10.1007/S001670100245. 11819022

[bibr41-10711007221108078] Möller M Movin T Granhed H Lind K Faxén E Karlsson J. Acute rupture of tendon Achillis. A prospective randomised study of comparison between surgical and non-surgical treatment. J Bone Joint Surg Br. 2001;83(6):843-848. doi:10.1302/0301-620X.83B6.11676. 11521926

[bibr42-10711007221108078] Muthu S Ramakrishnan E. Fragility analysis of statistically significant outcomes of randomized control trials in spine surgery: a systematic review. Spine (Phila Pa 1976). 2021;46(3): 198-208. doi:10.1097/BRS.0000000000003645. 32756285

[bibr43-10711007221108078] Nilsson-Helander K Silbernagel K Thomeé R , et al. Acute achilles tendon rupture: a randomized, controlled study comparing surgical and nonsurgical treatments using validated outcome measures. Am J Sports Med. 2010;38(11):2186-2193. doi:10.1177/0363546510376052. 20802094

[bibr44-10711007221108078] Ochen Y Beks RB Heijl M van , et al. Operative treatment versus nonoperative treatment of Achilles tendon ruptures: systematic review and meta-analysis. BMJ. 2019;364:5120. doi:10.1136/BMJ.K5120. PMC6322065 30617123

[bibr45-10711007221108078] Olsson N Silbernagel K Eriksson B , et al. Stable surgical repair with accelerated rehabilitation versus nonsurgical treatment for acute Achilles tendon ruptures: a randomized controlled study. Am J Sports Med. 2013;41(12):2867-2876. doi:10.1177/0363546513503282. 24013347

[bibr46-10711007221108078] Otte WM Vinkers CH Habets P IJzendoorn DGP van Tijdink JK. Almost significant: trends and P values in the use of phrases describing marginally significant results in 567,758 randomized controlled trials published between 1990 and 2020. medRxiv. March 2021:2021.03.01.21252701. doi:10.1101/2021.03.01.21252701.

[bibr47-10711007221108078] Parisien RL Constant M Saltzman BM , et al. The fragility of statistical significance in cartilage restoration of the knee: a systematic review of randomized controlled trials. Cartilage. 2021;13(1_suppl):147S-155S. doi:10.1177/19476035211012458. PMC8808853 33969744

[bibr48-10711007221108078] Parisien RL Danford NC Jarin IJ Li X Trofa DP Vosseller JT. The fragility of statistical findings in achilles tendon injury research: a systematic review. J Am Acad Orthop Surg Glob Res Rev. 2021;5(9):e21.00018. doi:10.5435/JAAOSGLOBAL-D-21-00018. PMC8415978 34491976

[bibr49-10711007221108078] Parisien RL Dashe J Cronin PK Bhandari M Tornetta P. Statistical significance in trauma research: too unstable to trust? J Orthop Trauma. 2019;33(12):e466-e470. doi:10.1097/BOT.0000000000001595. 31356443

[bibr50-10711007221108078] Parisien RL Ehlers C Cusano A Tornetta P Li X Wang D. The statistical fragility of platelet-rich plasma in rotator cuff surgery: a systematic review and meta-analysis. Am J Sports Med. 2021;49(12):3437-3442. doi:10.1177/0363546521989976. 33646884

[bibr51-10711007221108078] Parisien RL Trofa DP Cronin PK , et al. Comparative studies in the shoulder literature lack statistical robustness: a fragility analysis. Arthrosc Sports Med Rehabil. 2021;3(6): e1899-e1904. doi:10.1016/J.ASMR.2021.08.017. PMC8689245 34977646

[bibr52-10711007221108078] Parisien RL Trofa DP Dashe J , et al. Statistical fragility and the role of P values in the sports medicine literature. J Am Acad Orthop Surg. 2019;27(7):e324-e329. doi:10.5435/JAAOS-D-17-00636. 30325880

[bibr53-10711007221108078] Parisien RL Trofa DP O’Connor M , et al. The fragility of significance in the hip arthroscopy literature: a systematic review. JB JS Open Access. 2021;6(4):e21.00035. doi:10.2106/JBJS.OA.21.00035. PMC8542173 34703967

[bibr54-10711007221108078] Renninger C Kuhn K Fellars T Youngblood S Bellamy J. Operative and nonoperative management of Achilles tendon ruptures in active duty military population. Foot Ankle Int. 2016;37(3):269-273. doi:10.1177/1071100715615322. 26537241

[bibr55-10711007221108078] Ruzbarsky J Khormaee S Daluiski A. The fragility index in hand surgery randomized controlled trials. J Hand Surg Am. 2019;44(8):698.e1-698.e7. doi:10.1016/J.JHSA.2018.10.005. 30420197

[bibr56-10711007221108078] Ruzbarsky J Khormaee S Rauck R Warren R. Fragility of randomized clinical trials of treatment of clavicular fractures. J Shoulder Elbow Surg. 2019;28(3):415-422. doi:10.1016/J.JSE.2018.11.039. 30771826

[57] Ruzbarsky J Rauck R Manzi J Khormaee S Jivanelli B Warren R. The fragility of findings of randomized controlled trials in shoulder and elbow surgery. J Shoulder Elbow Surg. 2019;28(12):2409-2417. doi:10.1016/J.JSE.2019.04.051. 31420227

[58] Slim K Nini E Forestier D Kwiatkowski F Panis Y Chipponi J. Methodological index for non-randomized studies (MINORS): development and validation of a new instrument. ANZ J Surg. 2003;73(9):712-716. doi:10.1046/j.1445-2197.2003.02748.x. 12956787

[59] Soroceanu A Sidhwa F Aarabi S Kaufman A Glazebrook M. Surgical versus nonsurgical treatment of acute Achilles tendon rupture: a meta-analysis of randomized trials. J Bone Joint Surg Am. 2012;94(23):2136-2143. doi:10.2106/JBJS.K.00917. 23224384 PMC3509775

[60] Sterne JAC Savović J Page MJ , et al. RoB 2: a revised tool for assessing risk of bias in randomised trials. BMJ. 2019; 366:l4898. doi:10.1136/BMJ.L4898. 31462531

[61] Twaddle B Poon P. Early motion for Achilles tendon ruptures: is surgery important? A randomized, prospective study. Am J Sports Med. 2007;35(12):2033-2038. doi:10.1177/0363546507307503. 17885221

[62] van der Linden-van der Zwaag H Nelissen R Sintenie J . Results of surgical versus non-surgical treatment of Achilles tendon rupture. Int Orthop. 2004;28(6):370-373. doi:10.1007/S00264-004-0575-9. 15241626 PMC3456894

[63] Walsh M Srinathan SK McAuley DF , et al. The statistical significance of randomized controlled trial results is frequently fragile: a case for a Fragility Index. J Clin Epidemiol. 2014;67(6):622-628. doi:10.1016/j.jclinepi.2013.10.019. 24508144

[64] Westin O Svensson Nilsson Helander K , et al. Cost-effectiveness analysis of surgical versus non-surgical management of acute Achilles tendon ruptures. Knee Surg Sports Traumatol Arthrosc. 2018;26(10):3074-3082. doi:10.1007/S00167-018-4953-Z. 29696317 10.1007/s00167-018-4953-z PMC6154020

[65] Willits K Amendola A Bryant D , et al. Operative versus nonoperative treatment of acute Achilles tendon ruptures: a multicenter randomized trial using accelerated functional rehabilitation. J Bone Joint Surg Am. 2010;92(17):2767-2775. doi:10.2106/JBJS.I.01401. 21037028

