# Supplementary material for: The Statistical Fragility of Operative vs Nonoperative Management for Achilles Tendon Rupture: A Systematic Review of Comparative Studies
Source: Foot Ankle Int. 2022 Aug 24;43(10):1331–9. doi: 10.1177/10711007221108078 (PMC9527367; doi:10.1177/10711007221108078)
Supplement: sj-docx-1-fai-10.1177_10711007221108078 – Supplemental material for The Statistical Fragility of Operative vs Nonoperative Management for Achilles Tendon Rupture: A Systematic Review of Comparative Studies [file sj-docx-1-fai-10.1177_10711007221108078.docx]

*Appendix 1: Studies meeting criteria.*

*FI and FQ in the first row for each study represents the mean value of all outcomes assessed in the study. All outcomes were measured as yes/no, except where indicated.*

** Represents reported values as a metric of “problems versus no problems” on EQ-5D-5L.*

*Abbreviations: FI, Fragility Index; FQ, Fragility Quotient; RCT, Randomized Control Trial; JBJS, Journal of Bone and Joint Surgery; Bone Joint J, Bone Joint Journal; FAI, Foot and Ankle International; AJSM, American Journal of Sports Medicine; KSSTA, Knee Surgery, Sports Traumatology, Arthroscopy; Int Orthop, International Orthopaedics; Y, Yes; N, No; MRI, Magnetic Resonance Imaging; LTF, Lost to Follow-up; DVT, Deep Venous Thrombosis.*

| Primary Author | Bergkvist ^3^ | Lim ^34^ | Renninger ^55^ | Gwynne-Jones ^20^ | Jaakkola ^26^ | Westin ^64^ | van der Linden-van der Zwaag ^35^ |
| --- | --- | --- | --- | --- | --- | --- | --- |
| A clearly stated aim | 2 | 2 | 2 | 2 | 2 | 2 | 2 |
| Inclusion of consecutive patients | 2 | 1 | 2 | 2 | 2 | 2 | 2 |
| Prospective collection of data | 0 | 0 | 0 | 0 | 0 | 0 | 0 |
| Endpoints appropriate to the aim of the study | 2 | 2 | 2 | 2 | 1 | 2 | 2 |
| Unbiased assessment of the study endpoint | 0 | 0 | 0 | 0 | 0 | 0 | 1 |
| Follow-up period appropriate to the aim of the study | 2 | 2 | 0 | 1 | 2 | 2 | 1 |
| Loss to follow-up less than 5% | 0 | 0 | 0 | 0 | 0 | 0 | 0 |
| Prospective calculation of the study size | 0 | 0 | 0 | 0 | 0 | 1 | 0 |
| An adequate control group | 2 | 2 | 2 | 2 | 2 | 2 | 2 |
| Contemporary groups | 2 | 0 | 1 | 2 | 2 | 2 | 1 |
| Baseline equivalence of groups | 1 | 2 | 2 | 0 | 1 | 1 | 1 |
| Adequate statistical analyses | 2 | 2 | 2 | 2 | 2 | 2 | 2 |
| Total MINORS score | 15 | 13 | 13 | 13 | 14 | 16 | 14 |
